# CAMKII Activation Is Not Required for Maintenance of Learning-Induced Enhancement of Neuronal Excitability

**DOI:** 10.1371/journal.pone.0004289

**Published:** 2009-01-28

**Authors:** Ori Liraz, Kobi Rosenblum, Edi Barkai

**Affiliations:** 1 Department of Neurobiology, Faculty of Sciences, Haifa University, Haifa, Israel; 2 Department of Biology, Faculty of Sciences, Haifa University, Haifa, Israel; Pennsylvania State University, United States of America

## Abstract

Pyramidal neurons in the piriform cortex from olfactory-discrimination trained rats show enhanced intrinsic neuronal excitability that lasts for several days after learning. Such enhanced intrinsic excitability is mediated by long-term reduction in the post-burst after-hyperpolarization (AHP) which is generated by repetitive spike firing. AHP reduction is due to decreased conductance of a calcium-dependent potassium current, the sI_AHP_. We have previously shown that learning-induced AHP reduction is maintained by persistent protein kinase C (PKC) and extracellular regulated kinase (ERK) activation. However, the molecular machinery underlying this long-lasting modulation of intrinsic excitability is yet to be fully described. Here we examine whether the CaMKII, which is known to be crucial in learning, memory and synaptic plasticity processes, is instrumental for the maintenance of learning-induced AHP reduction. KN93, that selectively blocks CaMKII autophosphorylation at Thr286, reduced the AHP in neurons from trained and control rat to the same extent. Consequently, the differences in AHP amplitude and neuronal adaptation between neurons from trained rats and controls remained. Accordingly, the level of activated CaMKII was similar in pirifrom cortex samples taken form trained and control rats. Our data show that although CaMKII modulates the amplitude of AHP of pyramidal neurons in the piriform cortex, its activation is not required for maintaining learning-induced enhancement of neuronal excitability.

## Introduction

Learning-induced cellular changes can be divided into two general groups: modifications that occur at synapses and modifications in the intrinsic properties of the neurons. While it is commonly agreed that changes in strength of connections between neurons in the relevant networks underlie memory storage, evidence have been mounting rapidly that also modifications in intrinsic neuronal properties, manifested as enhanced neuronal excitability, underlie specific stages of learning (for reviews see [1]–[3].

Learning induced enhancement in neuronal excitability was shown in hippocampal neurons following classical conditioning [4]–[5], water-maze training (Oh et al., 2003), and in piriform cortex neurons following olfactory-discrimination (OD) learning (1. This enhanced excitability is manifested by reduced spike frequency adaptation [4], [6], and lasts for several days after training completion [1].

Neuronal adaptation is modulated by the post-burst after-hyperpolarization (AHP), generated by potassium currents which develop following spike firing [6]–[9]. The post-burst AHP is reduced after learning, [10]. Indeed, several studies indicate that the learning-induced reduction in neuronal adaptation and in AHP amplitude results from reduction in a Ca^2+^-dependent potassium current [9], [ 11]–[12]. It was recently shown that only the apamin-insensitive portion of the post-burst AHP is reduced after olfactory-discrimination learning [13], suggesting that learning modulates specifically the sI_AHP_. Changes in the sI_AHP_ were also implicated in learning-related modifications in hippocampal neurons after spatial learning [14].

While it has been shown that olfactory-learning induced AHP reduction in piriform cortex neurons is maintained by persistent activation of both PKC and ERK [15], [16], it is still to be determined whether other key second messenger systems are also instrumental is such prolonged modulation of neuronal excitability.

Calcium/calmodulin-dependent kinase II (CaMKII) has been implicated in learning [17], [18]. Although it is usually thought to affect synaptic plasticity, a recent study suggests that it may be also involved in modulating learning-relevant reduction in the post burst AHP, by restricting the post-burst AHP reduction to learning-specific processes only [19]. Autophosphorylation of CaMKII is a particularly a potential mechanism by which this protein may obtain a long lasting effect on the calcium activated potassium current that mediate the post-burst AHP.

The purpose of the present study was to examine whether CaMKII affects the post-burst AHP in piriform cortex neurons, and whether its persistent activation is required for prolonged maintenance of learning-induced enhancement of neuronal excitability.

## Results

Recordings in neurons from trained and pseudo-trained rats were performed three days after the last training session, when learning-related reduction in AHP amplitude is most prominent [1]. As previously reported [6], [10], [13], in control saline Ringer solution (NSR), the averaged post-burst AHP in neurons from trained rats was significantly smaller compared with the averaged AHP amplitudes in neurons from the control group (pseudo-trained and naive rats); the averaged peak amplitude of neurons from the control group (in mV) was 8.47±2.9 (n = 15) and in neurons form trained rats 6.58+1.14 (n = 6).

### Learning does not modify the effect of CaMKII on the post burst AHP

**Figure 1:**
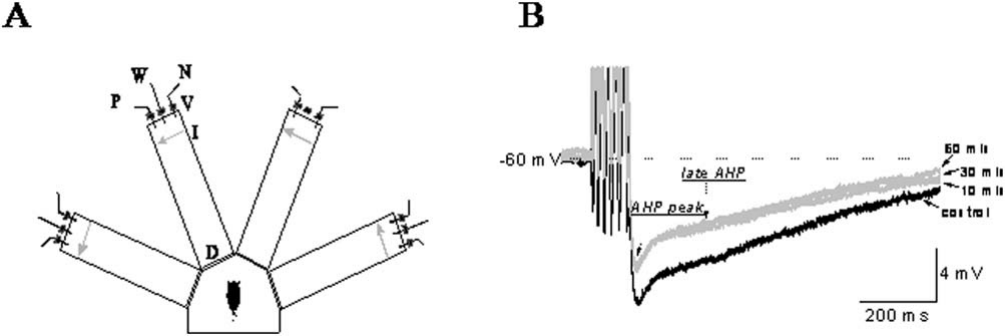
training apparatus and AHP measurements. A. Schematic description of the 4-arm maze. Protocols for trained and pseudo- trained rats are similar: an electronic ‘start’ command opens randomly two out of eight valves (V), releasing a positive-cue odor (P) into one of the arms and a negative-cue odor (N) into another. Eight seconds later, the two corresponding guillotine doors (D) are lifted to allow the rat to enter the selected arms. Upon reaching the far end of an arm (90 cm long), the rat body interrupts an infrared beam (I, arrow) and a drop of drinking water is released from a water hose (W) into a small drinking well (for a trained rat - only if the arm contains the positive-cue odor, for pseudo- trained rat- randomly). A trial ends when the rat interrupts a beam, or in 10 seconds, if no beam is interrupted. A fan is operated for 15 seconds between trials, to remove odors. B. Current clamp recordings for AHP measurements in a piriform cortex neuron. The holding membrane potential is −60 mV and the AHP is generated by 100 ms depolarizing current step injection via the recording electrode, to generate a train of 6 action potentials. Amplitude of the post-burst AHP was measured at two time points; at its peak (arrow marked as AHP peak) and 200 msec after termination of the depolarizing pulse (arrow marked as late AHP). KN93 application reduced the considerably the AHP amplitude. This reduction usually reached its almost maximal effect after 10 min. arrows indicate time after KN93 application.

**Figure 2:**
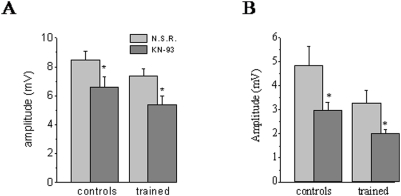
CaMKII inhibtor's effect on the post burst AHP is not modified after learning. KN93 reduced significantly the AHP amplitude at the peak of the AHP (A) and 200 msec after termination of the depolarizing pulse (B). Consequently the difference between the groups remained significant (p<0.05) at the two time points. The post-burst AHP amplitude was measured in each neuron before and at least 30 min after KN93 application. AHP was measured in 15 neurons from 11 control rats and 6 neurons taken for 5 trained rats. Values represent mean±SE.

To examine whether the long-lasting reduction in post burst AHP amplitude is maintained by CaMKII activation, we applied its specific inhibitor, KN93 (10 µM). A strong effect of KN93 on the AHP amplitude was apparent within 10 min after the beginning of application (Figure 1B). This relatively short exposure time was sufficient to induce a maximal effect; further exposure for up to one hour did not result with further AHP reduction (figure 1B). Thus, in each cell the AHP amplitude measurements were performed prior to and 30 min after KN93 application. KN93 decreased significantly the amplitude of the post burst AHP at the two time point at which it was measured. At the peak of the post burst AHP, the amplitude in neurons from the control group was reduced significantly to 7.37±1.9 mV and in neurons from trained rats to 5.36+1.6 mV (figure 2A). These values correspond to an averaged decrease of 11.4% in the peak amplitude for control neurons and 9.9% for trained neurons. Consequently, after KN93 application, the post burst AHP was still significantly lower in neurons from trained rats (P<0.05). In most piriform cortex pyramidal neurons, the peak of the post burst AHP is mediated by two calcium activated potassium currents, the I_AHP_ and the sI_AHP_ [13]. To examine whether the effect of CaMKII on the slower sI_AHP_ is modified after learning, we measured its effect on the AHP amplitude at the delay of 200 msec after termination of the depolarizing pulse (see figure 1B). We found that at this time point the effect of KN93 is indeed more pronounced. However, it was similar for neurons from trained rats and controls. At this time point, the amplitude of the AHP in neurons from the control group was reduced significantly from 4.83+3.3 to 3.3+2.0 (p<0.01), and in neurons from trained rats from 2.97+0.8 to 2.02+0.4 (p<0.05) (figure 2A). These values correspond to an averaged decrease of 30.5% in the AHP amplitude for control neurons and 30.9% for trained neurons. Thus, while the effect of the CaMKII blocker is much more pronounced on the late AHP, it is not modified after learning.

### Olfactory-discrimination learning is not correlated with enhanced CaMKII activation in the piriform cortex

**Figure 3:**
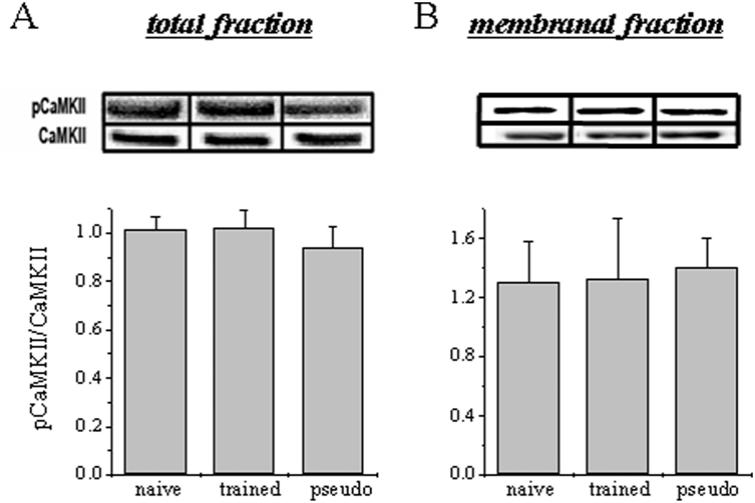
CaMKII phosphorylation level is not modified after learning. A. After learning the level of phosphorylated (Thr286) CaMKII in the total fraction is similar in all groups. The protein level is normalized to the average value obtained from the naïve animals. Summarized data are presented as mean O.D.±SE, data was taken from 11 naïve, 11 trained and 12 pseudo trained rats. Above are representative immunoblots for CaMKII protein and pCaMKII prepared from the piriform cortex of naïve, pseudo-trained and trained animals. B. The the level of phosphorylated (Thr286) CaMKII in the membranal fraction is also similar in all groups. The protein level is normalized to the average value obtained from the naïve animals. Summarized data are presented as mean O.D.±SE, data was taken from 4 naïve, 5 trained and 5 pseudo trained rats. Above are representative immunoblots for CaMKII protein and pCaMKII prepared from the piriform cortex of naïve, pseudo-trained and trained animals.

Since learning-induced enhancement of post-burst AHP reduction is independent of persistent CaMKII activation, we hypothesized that learning should not be accompanied by an increase in CaMKII phosphorylation at extra synaptic sites. Using western blot analysis, we measured the total amount of CaMKII and its phosphorylation state in the three experimental groups. We found the level of phosphorylated CaMKII (at Thr286) for the total homogenate fraction is not modified after learning. The total amount of CaMKII protein for the three groups were: naïve 1.01±0.05, pseudo-trained 0.94±0.09, trained 1.02±0.08. The pCaMKII/CaMKII ratio was also similar for the three groups; naïve 1.01±0.16, pseudo-trained 0.94±0.25, trained 1.01±0.07 (figure 3A).

We further examined whether a learning-induced increase in CaMKII phosphorylation occurs, but is confined to membranal fraction only. We found the level of phosphorylated CaMKII for the membranal fraction is not modified after learning. The total amount of CaMKII protein for the three groups were: naïve 1.00±0.12, pseudo-trained 1.08±0.13, trained 0.92±0.08. The pCaMKII/CaMKII ratio was also similar for the three groups; (naïve 1.31±0.55, pseudo-trained 1.41±0.43, trained 1.33±0.83) (figure 3B).

These results further suggest that CaMKII activation or modulation of expression is not necessary for the maintenance of learning-relevant enhancement in neuronal excitability.

## Discussion

CaMKII was shown to have a pivotal role in learning [17], [18]. In particular, it has a key role in memory formation and consolidation [20], [21], but not in the maintenance of long-term memory [21], [22]. At the sub-cellular level, CaMKII has been shown to control synaptic plasticity by affecting both types of ionotropic glutamate receptors, AMPA [23] and NMDA [24]. Although to date the role of CaMKII in memory formation and maintenance was mostly thought to occur via its effects on synaptic transmission, it may also act on intrinsic neuronal properties. Such a dual role in olfactory learning was also shown for other second messenger systems [15], [16].

We have previously shown that learning-induced long-lasting post-burst AHP reduction is maintained by persistent activation of PKC and ERK [15], [16]. Moreover, ERK was found to be activated in the piriform cortex in correlation with olfactory-discrimination training [16]. Here we show that CaMKII has a profound effect the amplitude of the late AHP in piriform cortex pyramidal neurons. Application of its blocker reduced the AHP by 30%. However, while blocking CaMKII autophosphorylation reduces the amplitude of the late AHP, its effect is not occluded by learning, suggesting that its long-term phosphorylation and activation is not involved in learning-related enhanced neuronal excitability. Such an effect is comparable to that previously shown for PKA which also reduces the AHP in the same extent, and effect that is also not modulated by learning [15]. In addition, CaMKII protein expression and its phosphorylated levels where not modified after learning, further suggesting that CaMKII is not involved in the maintenance of learning-relevant AHP modulation

Using knockout mice, in which autophosphorylation CaMKII is prevented, Ohno and his colleagues [19] showed that CaMKII is not required for post-burst AHP reduction in CA1 hippocampal neurons. However, it is necessary for this reduction to occur in a learning-specific manner only. Notably these knockout mice had learning-deficit in the Morris water maze task, but this can be well attributed to CaMKII's effect on synaptic plasticity.

Several studies indicate that the learning-induced reduction in neuronal adaptation and in AHP amplitude results from reduction in one or more Ca^2+^-dependent potassium currents [6], [11], [12]. Evidences suggest that changes in the sI_AHP_ may account for learning-related modifications in neuronal excitability in hippocampal [12] and pirifom cortical neurons [13]. The post-burst AHP in piriform cortex pyramidal neurons is modulated by acetylcholine and noradrenaline [11], [13]. To date, four messenger systems (PKA, PKC, ERK and here CaMKII) where shown to modulate the post-burst AHP in these neurons [15], [16]. However, the effects of PKA and CaMKII on the post burst AHP remain stable after learning.

Taken together these data strongly indicate that while AHP can be modulated by different signal transduction second messenger cascades, the long-lasting AHP reduction following learning is maintained specifically by PKC and ERK signal transduction pathways. It is yet to be determined how these signal transduction cascades are modified for such a long time to enable prolonged changes in neuronal excitability. One particularly interesting suggestions is that persistent activation of the kainases in the cascade may form a positive feedback loop of persistent activation, that could counteract the lost of activity due to the protein turnover and signal withdrawn. Such a system has the potential to store information [25], [26]. Although such persistent activation is implicated in long-lasting synaptic enhancement, it could potentially support also long-term modulation of intrinsic neuronal excitability.

## Materials and Methods

### Animal training

#### Subjects and apparatus

Age-matched young adult Sprague-Dawly male rats were used. Prior to training they were maintained on a 23.5 hr water-deprivation schedule, with food available *ad libitum*. Olfactory discrimination training protocol was performed daily on each trained and pseudo-trained rat in a 4-arm radial maze (figure 1A), with commercial odors that are regularly used in the cosmetics and food industry. Behavioral experiments were approved by the university of Haifa institutional committee for animal experiments, in accordance to the NIH guidelines.

#### Training

Olfactory training consisted of 20 trials per day for each rat as previously described (Saar et al., 2001). In short, in each trial the rat had to choose between two odors (positive- and negative-cue) presented simultaneously. Rats designated to the trained group were rewarded upon choosing the positive cue. Rats in the pseudo-trained group were rewarded in a random fashion, upon choosing any odor. The criterion for learning was at least 80% positive-cue choices in the last 10 trials of a training day, as was previously used [6], [27], [28]. Rats in the naive group were water restricted, but not exposed to the maze. Typically, 2–3 trained rats and 2–3 pseudo-trained rats were trained at the same training period, and all the rats in the trained group had to meet the criteria for the first pair of odors before all trained and pseudo trained rats were exposed to a second pair of odors. Training for a new pair began only after training for the second pair was completed for all rats. Rats were trained to discriminate between 3 pairs of odors, to confirm that rule learning was achieved and to ensure that rats are always sacrificed three days after the last training session. As previously described [6], [28], rats indeed learned the second and third pairs of odors much faster than the first pair (7–8 days of training for the first pair and 1–2 days for the second and third pairs). A detailed description of OD learning efficacy is described elsewhere [28].

### Slice preparation, stimulation and recording

400 µm coronal brain slices were cut as previously described [10] and kept in oxygenated (95% O_2_+5% CO_2_) normal saline Ringers' (NSR) solution (in mM: NaCl 124, KCl 3, MgSO_4_ 2, NaH_2_PO_4_ 1.25, NaHCO_3_ 26, CaCl_2_ 2 and glucose 10). Intracellular recordings were obtained from pyramidal cells in layer II of the piriform cortex, with 4M K-Acetate-filled sharp glass microelectrodes, at 35°C. Several piriform cortex slices were obtained from each rat.

AHPs were recorded within minutes after good recording conditions were established (resting potential of at least −65 mV and action potential amplitude of 80 mV or more). To standardize AHP recordings, neurons were depolarized to holding potential of −60 mV by direct current application via the recording electrode. Post-burst AHP amplitude was then measured following a 100 ms depolarizing current step with intensity that generates 6 action potentials. The AHP amplitude was determined from average of 8 consecutive measurements of AHP evoked in responses to stimuli applied once every 10 seconds.

The post-burst AHP amplitude was measured at two points. The first at the peak of the hyperpolarizing voltage deflection that follows an evoked train of six action potentials (figure 1B) and the second at delay of 200 msec after pulse offset. In these neurons, the peak post-burst AHP amplitude is usually composed of both the I_AHP_ and the sI_AHP_, while the later time point reflects the sI_AHP_ current only [13]. The stimulus intensity required to evoke 6 action potentials always remained constant throughout the recording period of each neuron, before and after drug application. Such stability of the required stimulus intensity well coincides with the stability of basic membrane properties, such as the neurons' input resistance or resting potential, which are not affected by the drugs.

The identity of the rat from which neurons were recorded (naive, trained, or pseudo-trained) was not known to the person conducting the experiments and measurements.

#### Kn93 and Kn92 application

The CaMKII inhibitor Kn93, and its inactive isomer Kn92, were applied via to the perfusing Ringer solution, at the concentration of 10 µM. All cells were recorded before and 30 min after drug application. Usually a maximal effect of Kn93 was observed within 10 min after beginning of application. Application of the inactive form of this inhibitor, Kn92 had no effect on five control neurons, on which it was tested.

### Immunoblot analysis

Rats were trained in olfactory discrimination task and were sacrificed 3 days after the acquisition of the third pair of odors. After decapitation and extraction of the brain from the skull, the piriform cortex was dissected out; Fresh piriform cortex was transferred into glass/glass 5 ml tissue grinder and added with 4 ml. homogenization buffer. All tissues are homogenized using 20 even strokes. Homogenization buffer: 10 mM HEPES, 2 mM EDTA, 2 mM EGTA, 0.5 mM DTT, 0.1 mM sodium orthovanadate Phosphatase inhibitor cocktail 1&2 (sigma), Protease inhibitor (sigma), 0.1 mM PMSF. SDS sample buffer: 2 ml Glycerol, 2 ml SDS 20%, 2.4 ml 0.5 Tris-HCL pH 6.8, 2.6 ml ddw, and 1 ml beta-mercaptoethanol. SDS sample buffer was added and following protein quantification using the Bradford reagent method all samples were equalize by tris-glycerol, boiled for 5 minutes and stored at −20°C.

10 ug of protein were loaded to a 12% gel and were transferred to a nitrocellulose membrane. Membranes were bloted with antibodies for CaMKIIα (1;1000) or p-CaMKIIα Thr 286 (1∶1000) that were purchased from Santa Cruz (Santa Cruz, California, USA). Goat anti-mouse (IgG) horseradish peroxidase (HRP) conjugated was from Jackson Immunoresearch (West Grove, PA, USA) and the enhanced chemiluminescense (ECL+) kit from Amersham (Piscataway, NJ, USA). For each gel we performed a cross bloting with the second antibody after striping. Results for both orders were indistinguishable. Quantification was performed using a CCD camera (ChemiDoc XRS -BioRad) and image analyzer (quantity one - BioRad). Each sample was measured relative to the background and phosphorylation levels were calculated as the ratio between the results from the antibody directed against the phospho-proteins divided by the antibody directed against the phosphorylation state-independent form of the proteins. Protein phosphorylation (phospho-protein/protein) is expressed as the ratio between trained and naive values. Values of 1 indicate no difference in protein phosphorylation following behavioral manipulations, while values different from 1 indicate an increase or decrease in phosphorylation. Membranal fraction was prepared as previously described [16].

### Statistical analysis

For all the electrophysiological measurements, between-groups comparison was done using one-way ANOVA for the three groups (naive, trained and pseudo-trained), and post-hoc multiple t-tests were than applied to compare between each two groups. The effect of Kn93 on each neuron was evaluated using paired t-test. Values throughout the text are presented as mean±SD. Data in graphs are presented as mean±SE.
